# Supplementary material for: Method for Classifying Behavior of Livestock on Fenced Temperate Rangeland in Northern China
Source: Sensors (Basel). 2019 Dec 3;19(23):5334. doi: 10.3390/s19235334 (PMC6928611; doi:10.3390/s19235334)
Supplement: Supplementary file 1 [file sensors-19-05334-s001.pdf]

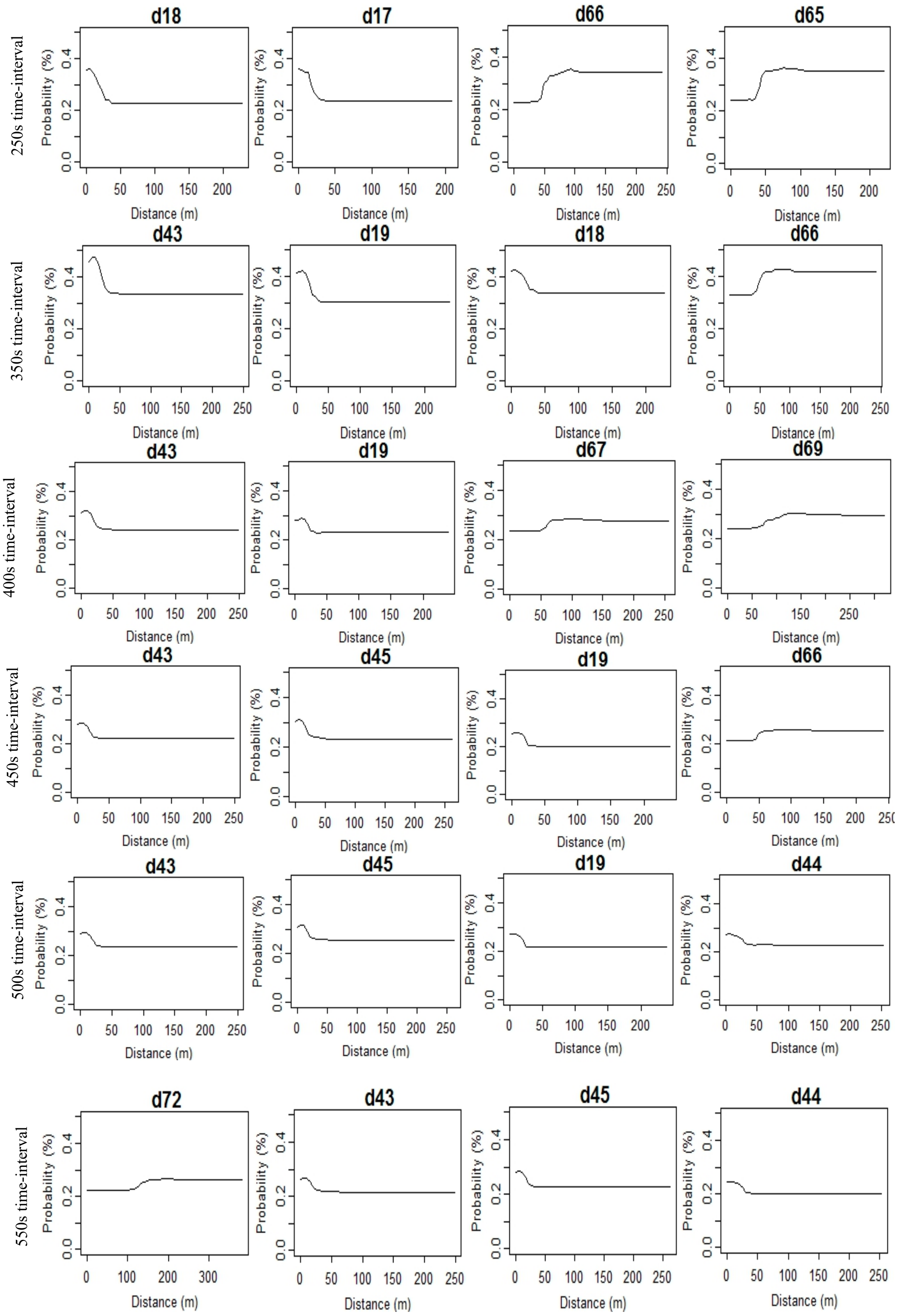
250s time-interval
d18
d17
d66
d65
350s time-interval
d43
d19
d18
d66
400s time-interval
d43
d19
d67
d69
450s time-interval
d43
d45
d19
d66
500s time-interval
d43
d45
d19
d44
550s time-interval
d72
d43
d45
d44
Probability (%)
Distance (m)

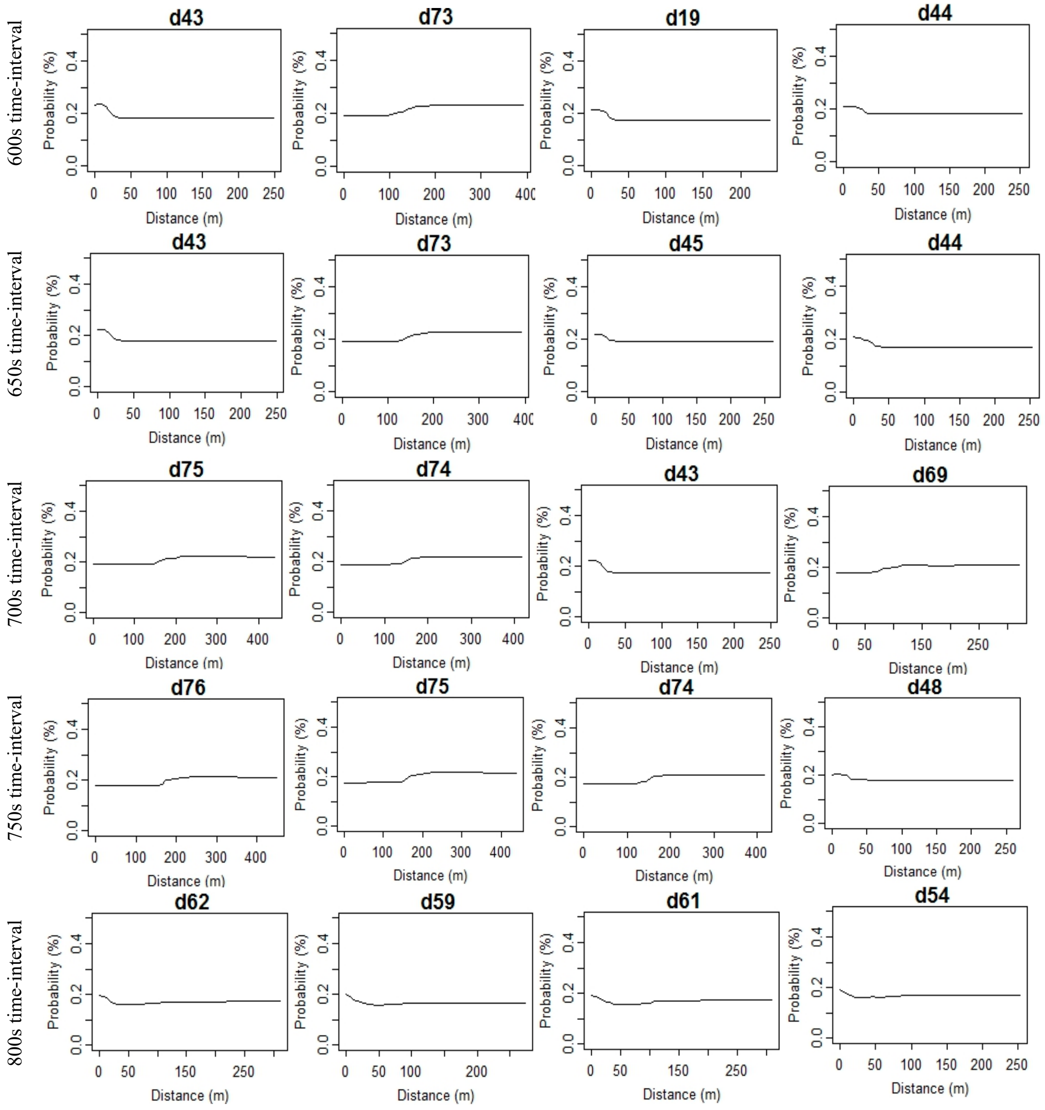

Figure S1 Partial dependence plots of non-grazing displayed first four importance variables from 250s to 800s time-interval in the GPS model
